# Caveolin-1 Regulation and Function in Mouse Uterus during Early Pregnancy and under Human In Vitro Decidualization

**DOI:** 10.3390/ijms23073699

**Published:** 2022-03-28

**Authors:** Zhuo Song, Bo Li, Mengyuan Li, Jiamei Luo, Yuqi Hong, Yuying He, Siting Chen, Zhenshan Yang, Chen Liang, Zengming Yang

**Affiliations:** College of Veterinary Medicine, South China Agricultural University, Guangzhou 510642, China; songzhuo.alex@gmail.com (Z.S.); boli373@163.com (B.L.); 17835422435@163.com (M.L.); scauluojm0126@163.com (J.L.); lhongyuqi@163.com (Y.H.); hheyuying@163.com (Y.H.); 15702094622@163.com (S.C.); le1730@126.com (Z.Y.); liangchen52@126.com (C.L.)

**Keywords:** Caveolin-1, decidualization, senescence, progesterone, estrogen, embryo, diabetes, insulin

## Abstract

Decidualization is essential to rodent and primate pregnancy. Senescence is increased during decidualization. Failure of senescence clearance during decidualization will cause pregnancy abnormality. Caveolin-1 is located in plasmalemmal caveolae and involved in senescence. However, whether caveolin-1 is involved in decidualization remains undefined. In this study, we examined the expression, regulation and function of Caveolin-1 during mouse early pregnancy and under mouse and human in vitro decidualization. From days 1 to 8 of pregnancy, Caveolin-1 signals are mainly located in endothelium and myometrium. Estrogen stimulates Caveolin-1 expression in endothelium. Deficiency of estrogen receptor α significantly promotes Caveolin-1 level in uterine stromal cells. Progesterone upregulates Caveolin-1 expression in luminal epithelium. During mouse in vitro decidualization, Caveolin-1 is significantly increased. However, Caveolin-1 is obviously decreased during human in vitro decidualization. Caveolin-1 overexpression and siRNA suppress and upregulate *IGFBP1* expression under in vitro decidualization, respectively. Blastocysts-derived tumor necrosis factor α (TNFα) and human Chorionic Gonadotropin (hCG) regulate Caveolin-1 in mouse and human decidual cells, respectively. Caveolin-1 levels are also regulated by high glucose and insulin. In conclusion, a low level of Caveolin-1 should be beneficial for human decidualization.

## 1. Introduction

During embryo implantation, embryos establish a close interaction with the maternal uterus and invade the endometrium to initiate decidualization in rodents and primates [1]. Decidualization is defined as the transformation of endometrial stromal cells into specialized secretory decidual cells, which can provide a nutritive and immuno privileged environment for embryo implantation and placental development [2]. Decidualization is critical for the establishment and maintenance of pregnancy [3]. *PRL* and *IGFBP-1* are now considered as widely used markers for human in vitro decidualization [2].

Cellular senescence is an irreversible cell cycle arrest and can be caused by various forms of cellular stresses [4]. The senescent cells remodel their chromatin and are resistant to stress [5]. The lysosomal enzyme senescence-associated β-galactosidase (SA-β-gal) is significantly increased during senescence and is the most used maker for senescence [6]. Decidualization starts with an acute cellular stress response and release of inflammatory mediators [7]. Recent data showed that an inflammatory phase coincides with the implantation window [8]. However, inflammatory stimulation of decidual cells also leads to the emergence of acute senescent cells [7]. During human in vitro decidualization, there is a significant increase of senescent cells [9].

In the plasma membrane of most cell types, caveolae are bulb-shaped and 50–100 nm wide cholesterol-rich lipid rafts [10]. Caveolin-1 is located in plasmalemmal caveolae and can modulate signaling pathways in many mammalian cells [11]. Caveolin-1 participates in a diversity of cellular and extracellular processes and plays important roles in tumorigenesis [12]. In fibroblasts, hydrogen peroxide or UV-C light can upregulate Caveolin-1 expression and induce premature senescence [13]. Caveolin-1 can suppress PI3K/AKT signaling pathway and lead to G0/G1 cell cycle arrest [10]. Because Caveolin-1 downregulation prevents stressor-induced premature senescence in NIH-3T3 cells and mouse embryonic fibroblasts, Caveolin-1 should be required for premature senescence [13,14]. However, Caveolin-1 deficient mice show aging-related phenotypes in various organs. Additionally, Caveolin-1 deficiency leads to mitochondrial dysfunction and premature senescence [15,16].

Although the mechanism underlying decidualization has been extensively studied, the expression, regulation, and function of Caveolin-1 during early pregnancy remain poorly defined. In this study, we showed that estrogen stimulated Caveolin-1 in endothelium and luminal epithelium and progesterone promoted Caveolin-1 in luminal epithelium and stroma. Blastocyst-derived factors regulated Caveolin-1 in decidual cells. Caveolin-1 downregulation should be beneficial for human decidualization.

## 2. Results

### 2.1. Caveolin-1 Expression during Early Pregnancy

**Figure 1:**
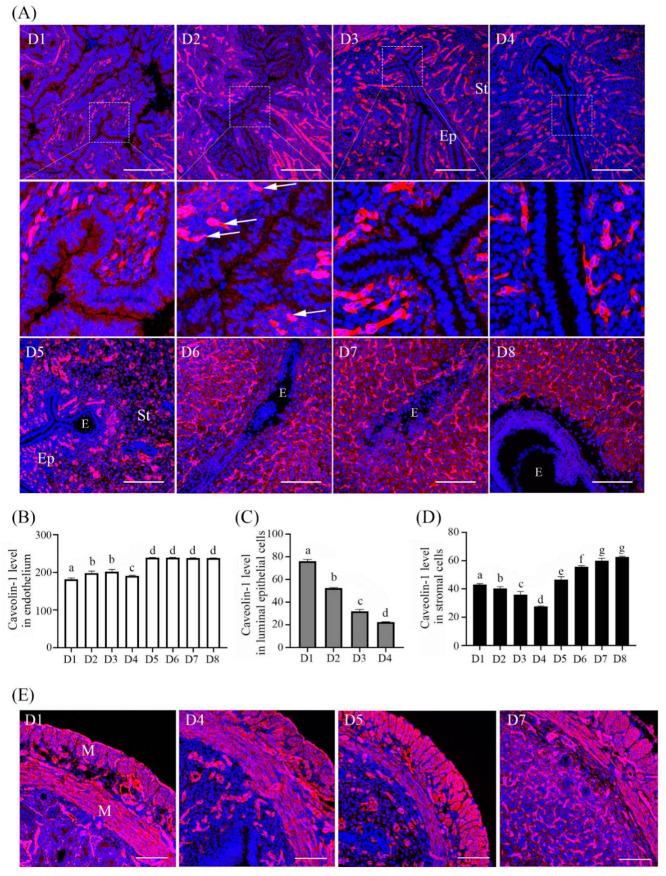
Caveolin-1 immunofluorescence in mouse uteri during early pregnancy. (**A**) Caveolin-1 immunofluorescence in mouse uteri from days 1 to 8. (**B**) Semi-quantitative Caveolin-1 level in endothelium. (**C**) Semi-quantitative Caveolin-1 level in luminal epithelial cells. (**D**) Semi-quantitative Caveolin-1 level in endometrial stromal cells. (**E**) Caveolin-1 immunofluorescence in mouse myometrium. Five mice per group. E, embryo; White arrow, endothelium; St, stromal cells; Ep, epithelium; M, muscle. Different letters on each bar show significant difference between two groups. Scale bar = 200 μm.

From days 1 to 8 of pregnancy, Caveolin-1 signal was mainly located in myometrium and endothelial cells (Figure 1A). Compared with day 1, Caveolin-1 signals in endothelium on days 2 and 3 were stronger. There was a high level of Caveolin-1 staining in endothelium from days 5 to 8 of pregnancy (Figure 1B). In endometrial stroma cells and luminal epithelial cells, Caveolin-1 signals gradually decreased from days 1 to 4 of pregnancy (Figure 1C,D). From days 5 to 8, Caveolin-1 signals in stromal cells gradually increased and were mainly located in the primary decidual zone (Figure 1D). The level of Caveolin-1 in myometrium remained relatively constant (Figure 1E).

### 2.2. Caveolin-1 Expression under Delayed Implantation and Activation

**Figure 2:**
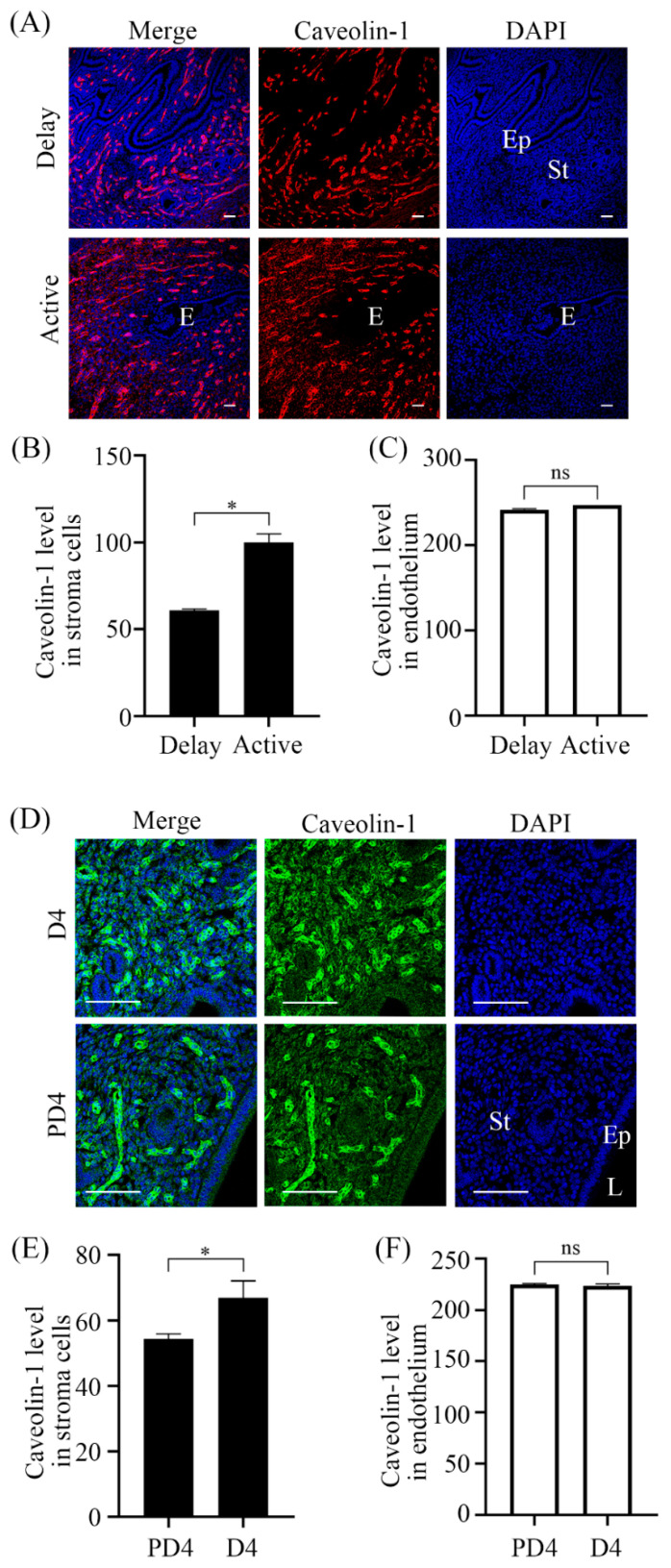
Caveolin-1 immunofluorescence in mouse uteri. (**A**) Mouse uteri under delayed implantation and activation, respectively. (**B**) Semi-quantitative Caveolin-1 level in endometrial stromal cells in (**A**). (**C**) Semi-quantitative Caveolin-1 level in endothelium in (**A**). (**D**) Mouse uteri on day 4 of pregnancy and day 4 of pseudopregnancy. (**E**) Semi-quantitative Caveolin-1 level in endometrial stromal cells in (**D**). (**F**) Semi-quantitative Caveolin-1 level in endothelium in (**D**). Five mice per group. E, embryo; St, stromal cells; Ep, epithelium; L, uterine lumen. *, *p* < 0.05; ns, not significant. Scale bar = 100 μm.

Because Caveolin-1 was strongly located in decidual cells, we asked whether Caveolin-1 expression was dependent on the presence of blastocysts. Under delayed implantation, Caveolin-1 signal was mainly located in endothelial cells (Figure 2A). After delayed implantation was activated by estrogen, the signals were significantly induced in endometrial stromal cells (Figure 2A,B) and remained unchanged in endothelium (Figure 2C). To further confirm effects of blastocysts on Caveolin-1 expression, Caveolin-1 expression in day 4 of pseudopregnancy was also examined (Figure 2D). Compared to day 4 of pregnancy, Caveolin-1 signal in stromal cells was significantly reduced in day 4 pseudo-pregnant uterus (Figure 2E), but Caveolin-1 signals in endothelium remained unchanged (Figure 2F).

### 2.3. Estrogen Regulation on Caveolin-1 Expression

**Figure 3:**
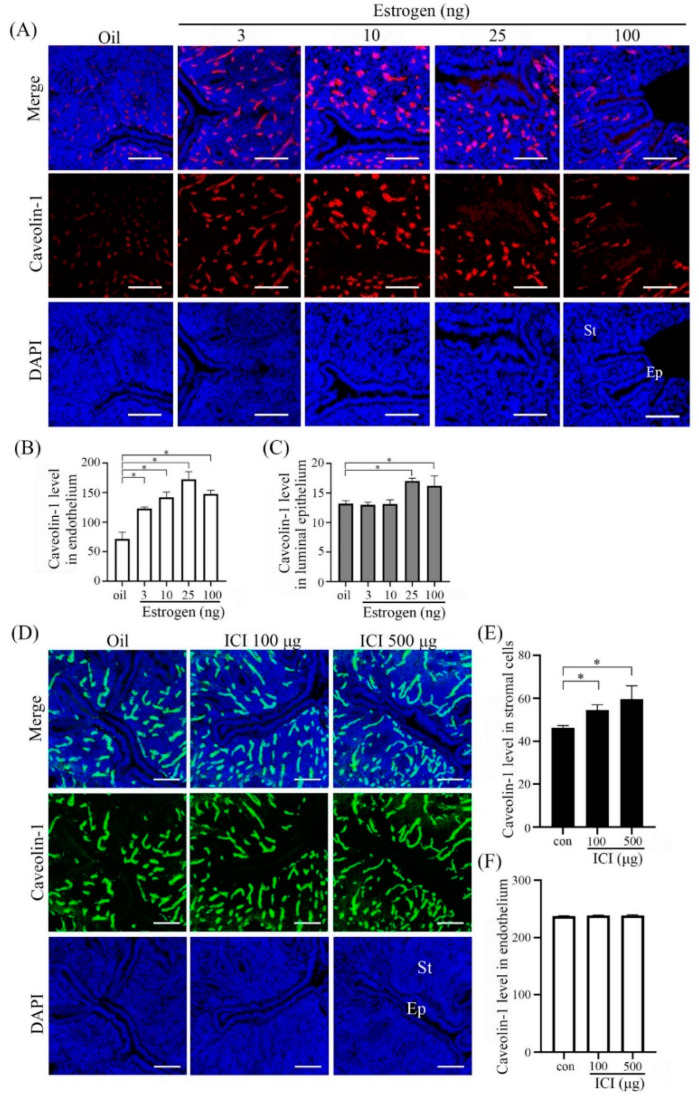
Caveolin-1 immunofluorescence in estrogen-treated uteri of ovariectomized mice. (**A**) Mouse uteri treated with different doses of estradiol-17β for 12 h. (**B**) Semi-quantitative Caveolin-1 level in endothelium in (**A**). (**C**) Semi-quantitative Caveolin-1 level in luminal epithelial cells in (**A**). (**D**) Mouse uteri treated with different doses of ICI 182,780 (ICI) for 12 h. (**E**) Semi-quantitative Caveolin-1 level in endometrial stromal cells in (**D**). (**F**) Semi-quantitative Caveolin-1 level in endothelium in (**D**). Five mice per group. St, stromal cells; Ep, epithelium; *, *p* < 0.05; Scale bar = 100 μm.

Because estrogen is essential for mouse early pregnancy [17], we tested the effects of estrogen on Caveolin-1 expression (Figure 3A). In ovariectomized mouse uteri, there was a weak Caveolin-1 signal in endothelial cells. When ovariectomized mice were treated with 3, 10, and 25 ng estradiol-17β, Caveolin-1 signals in endothelial cells were significantly increased, which was suppressed by 100 ng estradiol-17β (Figure 3B). Additionally, there was a weak Caveolin-1 signal in uterine epithelial cells when ovariectomized mice were treated with 25 and 100 ng estradiol-17β (Figure 3C). When ovariectomized mice were treated with 100 or 500 μg ICI 182,780 (Figure 3D), an antagonist of estrogen receptor, the levels of Cavelin-1 in stromal cells were slightly up-regulated (Figure 3E), but ICI 182,780 had no detectable effects on Caveolin-1 level in endothelial cells (Figure 3F).

**Figure 4:**
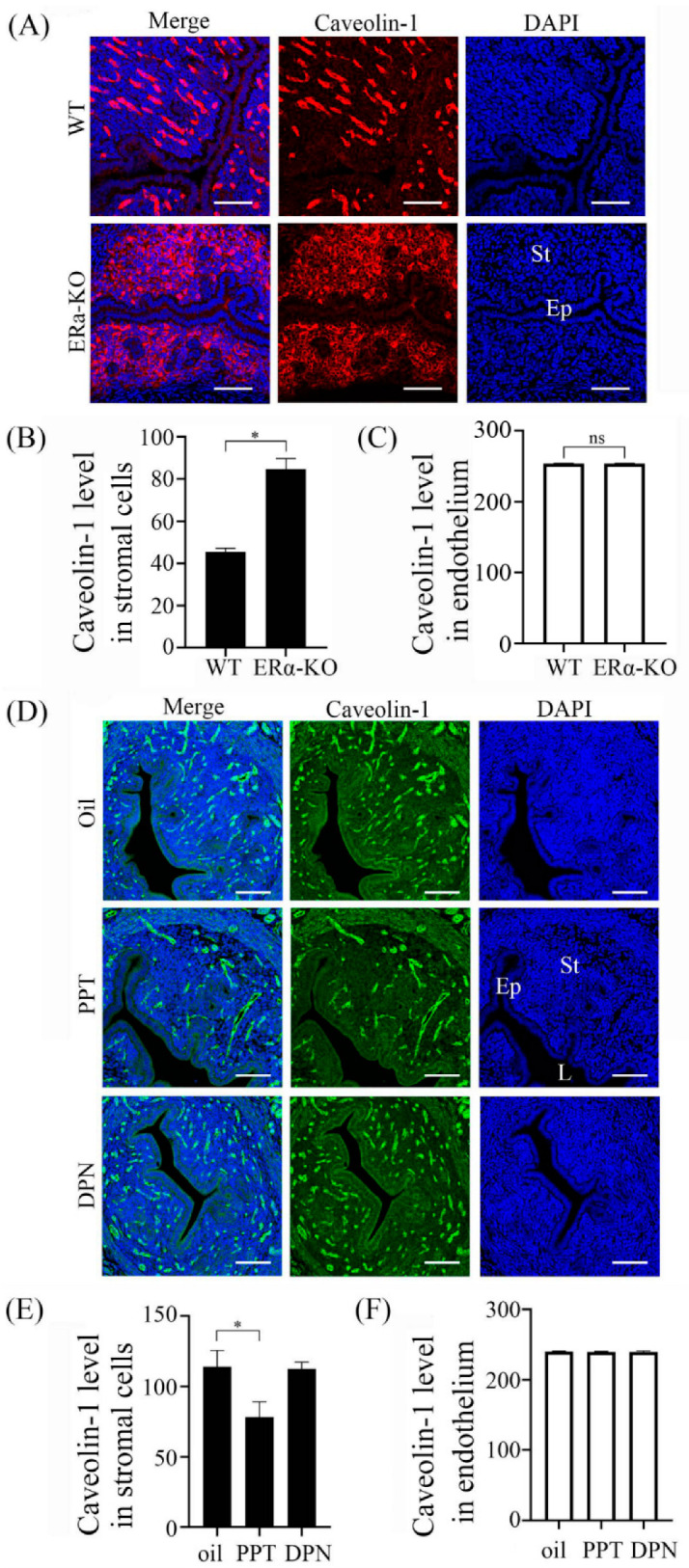
Caveolin-1 immunofluorescence in ovariectomized mouse uteri. (**A**) Mouse uteri from wild-type and ERα-KO mice, respectively. (**B**) Semi-quantitative Caveolin-1 level in endometrial stromal cells in (**A**). (**C**) Semi-quantitative Caveolin-1 level in endothelium in (**A**). (**D**) Mouse uteri treated with 100 mg PPT (agonist of estrogen receptor α) or 50 mg DPN (agonist of estrogen receptor β) and for 12 h. (**E**) Semi-quantitative Caveolin-1 level in endometrial stromal cells in (**D**). (**F**) Semi-quantitative Caveolin-1 level in endothelium in (**D**). Five mice per group. St, stromal cells; Ep, epithelium; L, uterine lumen; *, *p* < 0.05; ns, not significant; Scale bar = 100 um.

To further verify the dependency of Caveolin-1 expression on estrogen receptor, we examined Caveolin-1 expression in estrogen receptor α (ERα) -deficient mice. In wild-type mouse uteri, there were strong signals in endothelial cells and weak signals in epithelial cells and stromal cells (Figure 4A). In ERα-deficient mice, Caveolin-1 signals in stromal cells were significantly stimulated compared with wild-type mice (Figure 4B), but the level of Caveolin-1 in endothelium had no significant difference (Figure 4C). Caveolin-1 signals in stromal cells were further suppressed by PPT (Figure 4D,E), an ERα agonist. When ovariectomized mice were treated with Diarylpropionitrile (DPN), an ERβ agonist, DPN had no detectable effect on Caveolin-1 level (Figure 4D,F).

### 2.4. Progesterone Regulation of Caveolin-1 Expression

**Figure 5:**
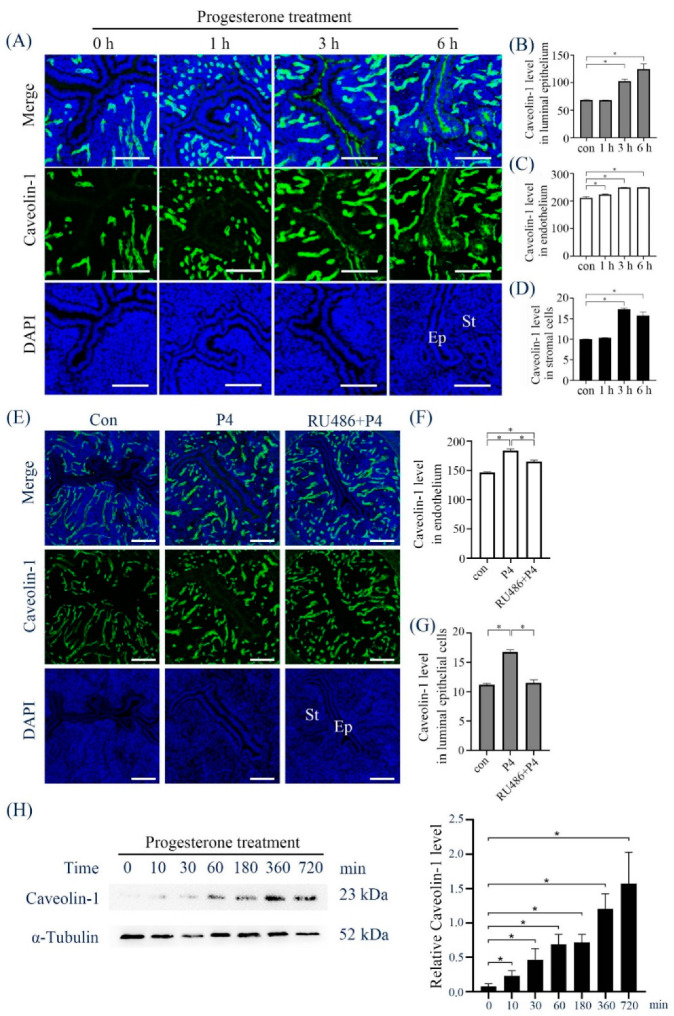
Caveolin-1 levels in progesterone-treated mouse uteri and stromal cells. (**A**) Caveolin-1 immunofluorescence in ovariectomized mouse uteri treated with progesterone for different time points. (**B**) Semi-quantitative Caveolin-1 level in luminal epithelial cells in (**A**). (**C**) Semi-quantitative Caveolin-1 level in endothelium in (**A**). (**D**) Semi-quantitative Caveolin-1 level in endometrial stromal cells in (**A**). (**E**) Caveolin-1 immunofluorescence in ovariectomized mouse uteri treated with progesterone or a combination of progesterone and RU486 for 12 h. (**F**) Semi-quantitative Caveolin-1 level in endothelium in (**E**). (**G**) Semi-quantitative Caveolin-1 level in luminal epithelial cells in (**E**). (**H**) Western blot and semi-quantitative analysis of Caveolin-1 protein in progesterone-treated mouse stromal cells. Five mice per group. *, *p* < 0.05; St, stromal cells; Ep, epithelium; Scale bar = 100 μm.

Progesterone is essential to the establishment and maintenance of pregnancy [18]. After ovariectomized mice were treated with progesterone for different time periods (Figure 5A), Caveolin-1 signals in luminal epithelial cells (Figure 5B), endothelial cells (Figure 5C), and stromal cells (Figure 5D) were stimulated at 3 and 6 h following progesterone treatment. Progesterone-induced Caveolin-1 signals in endometrial epithelial cells were abrogated by RU486, an antagonist of progesterone receptor (PR) (Figure 5E–G). Western blot analysis also confirmed that Caveolin-1 expression in stromal cells were increased by progesterone treatment at different time points (Figure 5H).

### 2.5. Role of Caveolin-1 during Mouse In Vitro Decidualization

**Figure 6:**
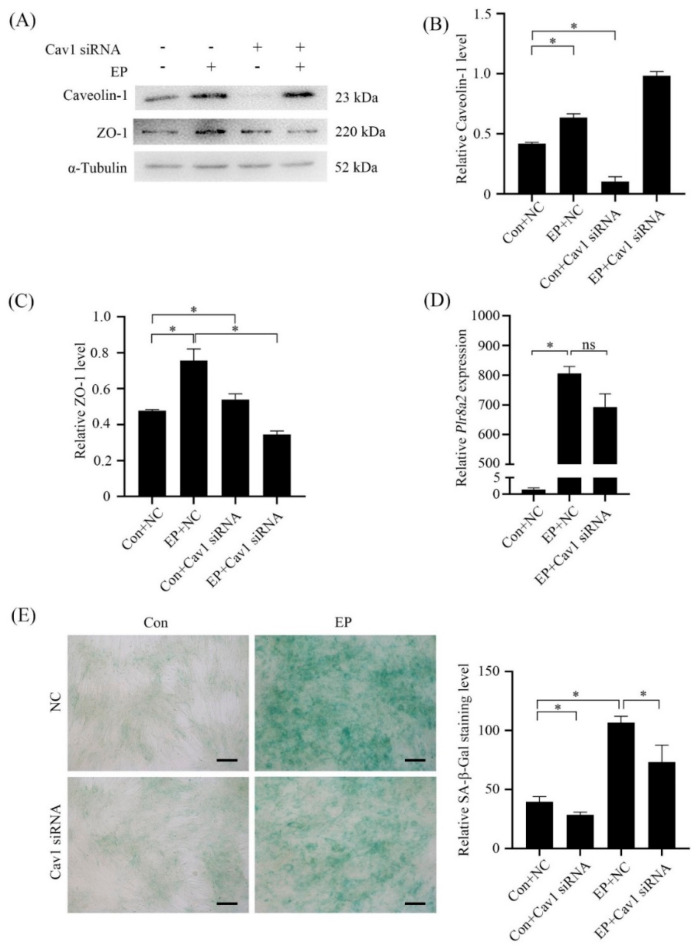
Effects of Caveolin-1 siRNA on mouse in vitro decidualization. (**A**) Western blot analysis of Caveolin-1 and ZO-1 proteins in stromal cells under in vitro decidualization for 2 days. (**B**) Semi-quantitative analysis of Caveolin-1 protein level in (**A**). (**C**) Semi-quantitative analysis of ZO-1 level in (**A**). (**D**) Real-time PCR analysis on the effect of Caveolin-1 siRNA on *Plr8a2* mRNA level in stromal cells under in vitro decidualization for 2 days. (**E**) SA-β-Gal staining and Semi-quantitative analysis in stromal cells under in vitro decidualization for 6 days. NC, negative control. *, *p* < 0.05; ns, not significant; Scale bar = 20 μm.

Because Caveolin-1 was expressed in decidual cells from days 5 to 8 of pregnancy, we examined role of Caveolin-1 during in vitro decidualization. The stromal-epithelial transition is a marker of decidualization [19]. Zonula occludens-1 (ZO-1) is a protein of tight junction and upregulated in mesenchymal-epithelial transition [20]. Under in vitro decidualization, Caveolin-1 level was significantly increased (Figure 6A,B). After Caveolin-1 expression was suppressed by Caveolin-1 siRNA, ZO-1 level was significantly increased compared with negative control (NC) (Figure 6A,C) although there was no detectable change on *Prl8a2* level, a marker for mouse in vitro decidualization (Figure 6D). Additionally, senescence is also induced during in vitro decidualization [9]. The activity of senescence-associated β-galactosidase (SA-β-Gal) was inhibited by Caveolin-1 siRNA under in vitro decidualization for 6 days. This result revealed that the expression of Caveolin-1 could influence the processing of senescence in mouse decidual cells (Figure 6E).

### 2.6. Caveolin-1 Expression under Human In Vitro Decidualization

**Figure 7:**
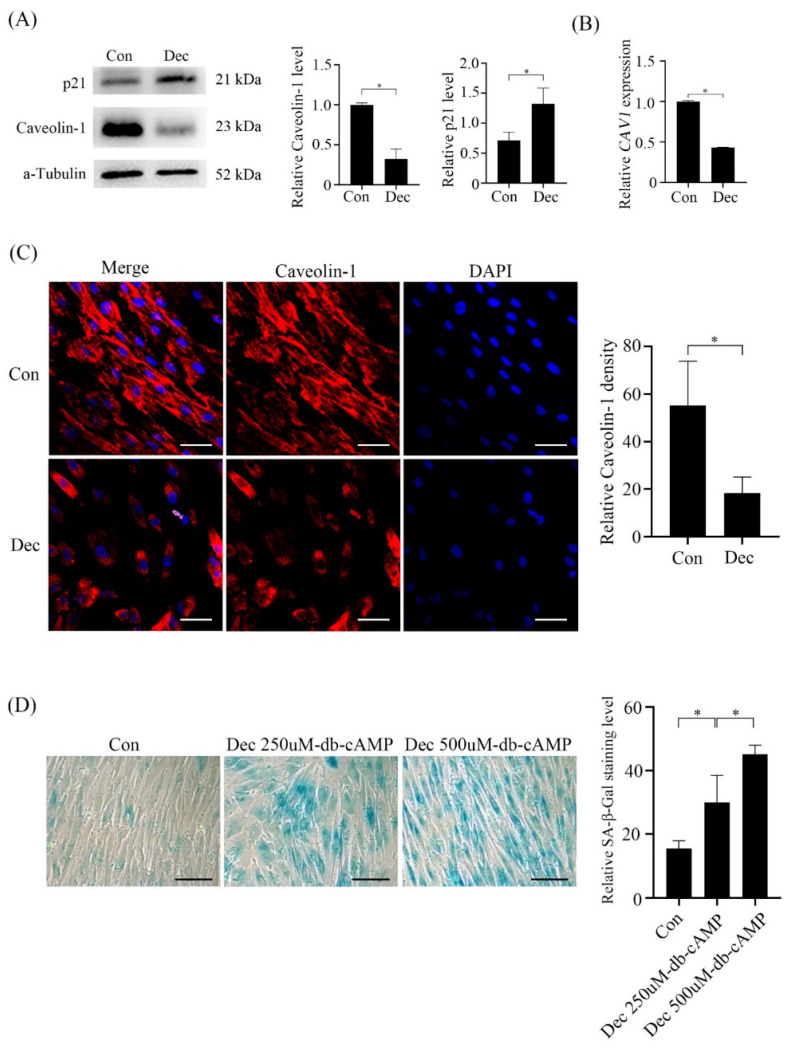
Caveolin-1 levels and SA-β-Gal staining in human stromal cells under in vitro decidualization. (**A**) Western blot analysis of Caveolin-1 and p21 protein levels in stromal cells under in vitro decidualization for 6 days. (**B**) Real-time PCR analysis of Caveolin-1 mRNA expression in stromal cells under in vitro decidualization for 4 days. (**C**) Caveolin-1 immunofluorescence and semi-quantitative analysis in stromal cells under in vitro decidualization for 6 days. (**D**) SA-β-Gal staining and semi-quantitative analysis in stromal cells under in vitro decidualization for 6 days with different doses of db-cAMP. Scale bar = 20 μm. *, *p* < 0.05.

Because Caveolin-1 was strongly expressed in mouse decidual cells, we wondered whether Caveolin-1 is expressed during human decidualization. Under in vitro decidualization, Caveolin-1 level was down-regulated, and p21 level was up-regulated, a marker for senescence (Figure 7A). Both qPCR and immunofluorescence confirmed the decrease of Caveolin-1 under in vitro decidualization (Figure 7B,C). The signals of SA-β-Gal activity were significantly increased under in vitro decidualization (Figure 7D).

### 2.7. Effects of Caveolin-1 Over-Expression on Human In Vitro Decidualization

**Figure 8:**
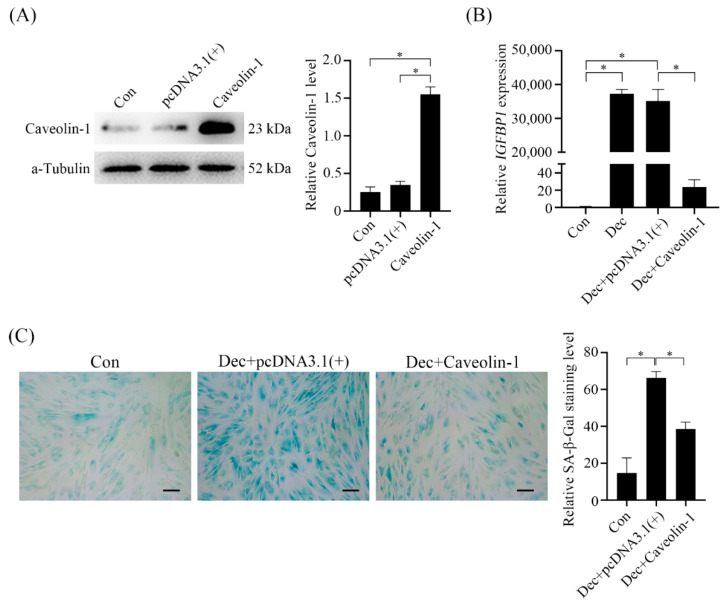
Effects of Caveolin-1 over-expression on human in vitro decidualization. (**A**) Western blot and semi-quantitative analysis of Caveolin-1 protein levels in Caveolin-1-overexpressed human stromal cells. (**B**) Real-time PCR analysis of *IGFBP1* mRNA expression in Caveolin-1-overexpressed stromal cells under in vitro decidualization. (**C**) SA-β-Gal staining and semi-quantitative analysis in Caveolin-1-overexpressed stromal cells under in vitro decidualization for 6 days. Scale bar = 20 μm. *, *p* < 0.05.

After Caveolin-1 level was significantly increased by Caveolin-1 overexpression (Figure 8A), there was a significant decrease of *IGFBP-1* expression (Figure 8B), a marker of human in vitro decidualization [19]. Caveolin-1 overexpression also caused a decrease of β-X-gal staining (Figure 8C).

**Figure 9:**
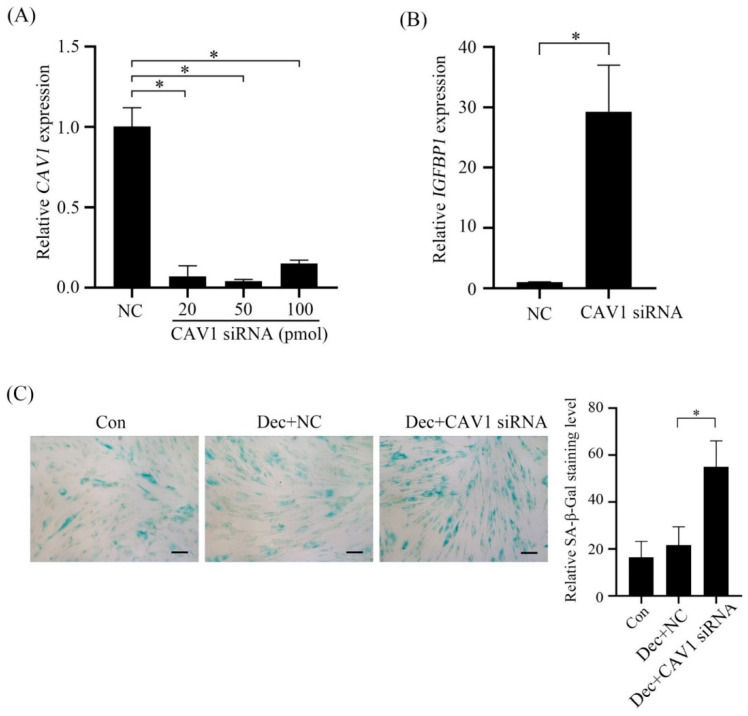
Effects of Caveolin-1 siRNA on human in vitro decidualization. (**A**) Real-time PCR analysis of Caveolin-1 mRNA expression after stromal cells were transfected with Caveolin-1 siRNA. (**B**) Real-time PCR analysis of IGFBP1 mRNA expression after stromal cells were transfected with Caveolin-1 siRNA. (**C**) SA-β-Gal staining in stromal cells after transfected with Caveolin-1 siRNA for 2 days. Scale bar = 20 μm. *, *p* < 0.05.

After Caveolin-1 was suppressed by Caveolin-1 siRNA (Figure 9A), there were significant increases of both *IGFBP-1* level and SA-β-Gal staining compared with negative control (Figure 9B,C).

### 2.8. Regulation of Caveolin-1 Expression

Our data showed that there was a big difference of Caveolin-1 expression in mouse uteri between day 4 pregnancy and day 4 pseudopregnancy, and between delayed implantation and activation, suggesting the involvement of blastocysts in regulating Caveolin-1 expression.

**Figure 10:**
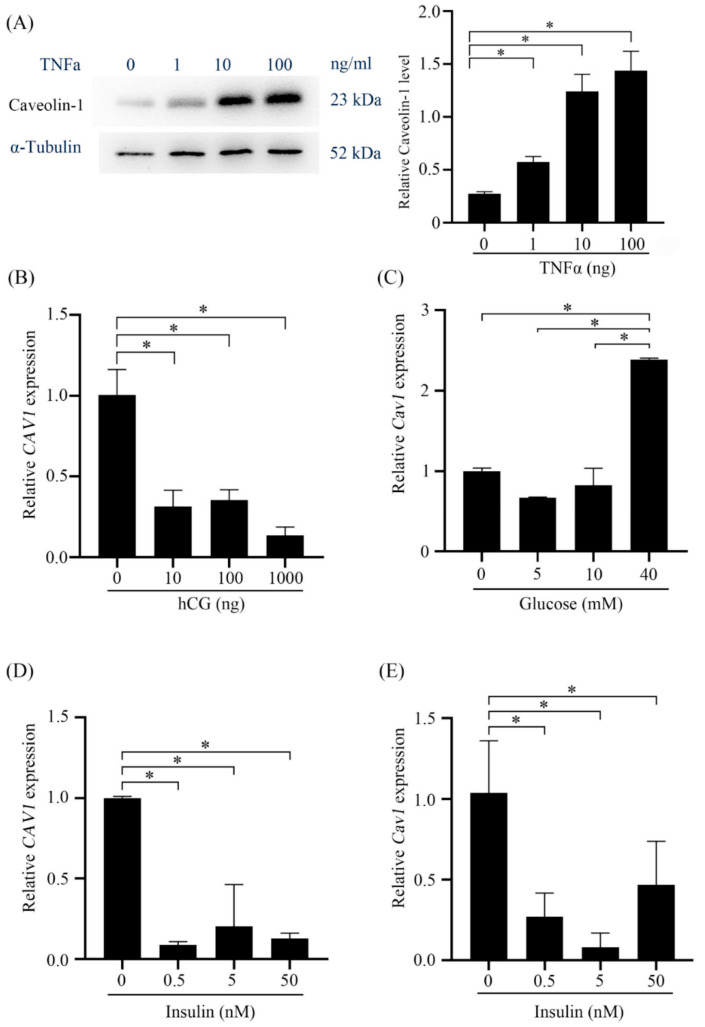
Regulation of Caveolin-1 by TNFα, hCG, high glucose, and insulin. (**A**) Western blots analysis of Caveolin-1 protein levels in mouse uterine stromal cells treated with TNFα for 30 min. (**B**) The levels of Caveolin-1 after human stromal cells were treated with hCG for 12 h. (**C**) The levels of Caveolin-1 in mouse stromal cells treated with glucose for 3 days. (**D**) The levels of Caveolin-1 in human stromal cells treated with insulin for 6 h. (**E**) The levels of Caveolin-1 in mouse stromal cells treated with insulin for 12 h. *, *p* < 0.05.

Compared to dormant blastocyst, tumor necrosis factor α (TNFα) is expressed in activated blastocysts [21]. When mouse stromal cells were treated with TNFα, Caveolin-1 levels were strongly stimulated (Figure 10A). Human chorionic gonadotropin (hCG) is strongly produced by blastocysts and syncytiotrophoblast [22,23]. hCG also promotes human decidualization [23]. When human stromal cells were treated with different dosages of hCG (10 ng, 100 ng, and 1 mg), Caveolin-1 level was significantly suppressed by hCG (Figure 10B).

In pancreatic *β*-cells, Caveolin-1 is involved in insulin secretion. Caveolin-1 knockdown can cause a significant increase of insulin secretion [10]. When stromal cells were treated with high-glucose, Caveolin-1 level was significantly increased (Figure 10C). However, insulin treatment inhibited the expression of Caveolin-1 both in human and mouse stromal cells (Figure 10D,E).

## 3. Discussion

In this study, Caveolin-1 signals were mainly localized in uterine myometrium and endothelium during moue early pregnancy. Caveolin-1 level in endothelium was stimulated by estrogen. ERα deficiency significantly upregulated Caveolin-1 in stromal cells. Under in vitro decidualization, Caveolin-1 protein level showed an opposite pattern between mice and humans. In humans, Caveolin-1 overexpression suppressed *IGFBP1* expression, while Caveolin-1 siRNA promoted *IGFBP1*.

### 3.1. Hormonal Regulation

In our study, Caveolin-1 was mainly localized in uterine myometrium and endothelium. A previous study also localized Caveolin-1 in endothelial cells and the myometrium in mouse uterus [24]. During rat early pregnancy, Caveolin-1 was localized at uterine epithelial cells and significantly increased at the time of implantation [25]. However, we did not detect Caveolin-1 signals in the luminal epithelium at implantation site on day 5 of pregnancy and estrogen-activated uterus.

We showed that Caveolin-1 was strongly detected in endothelial cells on days 2 and 3 of pregnancy, matched with the high level of estrogen at this period during early pregnancy [18]. In ovariectomized mouse uteri, estrogen also stimulated Caveolin-1 expression in endothelial cells [26]. In human breast cancer cell line BT474, estrogen increases the expression of Caveolin-1 [27]. In bovine aortic endothelial cells, both mRNA and protein for Caveolin-1 were increased significantly by estrogen treatment [28]. In human breast cancer MCF-7 cells, estrogen inhibits Caveolin-1 synthesis [29]. In neuronal cells lacking functional ERα, Caveolin-1 is upregulated. Ectopic expression of ERα causes the transcriptional suppression of Caveolin-1 [30]. In our study, Caveolin-1 signals in uterine stromal cells were significantly increased in ERα-deficient mouse uterus. In ovariectomized rat uterus, estrogen receptor (ERα) antagonist ICI 182,780 (1 mg/mouse) also increased the level of Caveolin-1 [31]. Additionally, Caveolin-1 also has a regulatory effect on ERα. ERα expression is significantly increased in Caveolin-1-deficient mammary epithelia [32]. In our study, Caveolin-1 signals in endothelial cells are stimulated by estrogen, but ERα deficiency causes a strong increase of Caveolin-1 signals in uterine stromal cells. It seems that the regulation of estrogen on Caveolin-1 level is dependent on cell types. During days 5 to 8 of pregnancy, Caveolin-1 was also strongly detected in decidual stromal cells. Because progesterone antagonizes estrogen action in mouse uterus [33], progesterone from corpus luteum may antagonize estrogen action to stimulate Caveolin-1 expression. In breast cancer line C4HD cells, caveolin-1 expression is upregulated by progestin [34]. We also showed that Caveolin-1 signals in luminal epithelial cells were upregulated by progesterone.

### 3.2. Senescence

During human in vitro decidualization, there is a significant increase of senescent cells [9]. In our study, SA-β-Gal staining was also increased under human in vitro decidualization. Caveolin-1 overexpression reduced the staining, while Caveolin-1 siRNA increased SA-β-Gal staining. Recent data showed that an inflammatory phase coincides with the implantation window [8]. However, inflammatory stimulation of decidual cells also lead to the emergence of acute senescent cells [7]. Recurrent pregnancy loss is associated with a pro-senescent decidual response during the peri-implantation window [35]. It is shown that reactive oxygen species (ROS) can induce premature cellular senescence. The nuclear erythroid 2 p45-related factor-2 (Nrf2) can mediate cytoprotective responses against oxidative stress. In colon cancer cells, Caveolin-1 can promote premature senescence through inhibiting Nrf2-dependent signaling [36]. Premature senescence is also compromised in Caveolin-1-deficient mouse embryonic fibroblasts [14]. However, in human fibroblasts, depletion of Caveolin-1 expression promotes primary cilia formation and induces premature senescence [37]. In unstimulated cells, either knockout or knockdown of Caveolin-1 induces senescence in resting human diploid fibroblasts [13]. Different from human in vitro decidualization, the SA-β-Gal staining under mouse in vitro decidualization is compromised by Caveolin-1 siRNA. It is possible that Caveolin-1 may contribute different roles in different cell types.

### 3.3. Decidualization

Under mouse in vitro decidualization, Caveolin-1 was significantly increased. However, Caveolin-1 was significantly downregulated under human in vitro decidualization. We also showed that under human in vitro decidualization, Caveolin-1 overexpression suppressed *IGFBP1* expression, while Caveolin-1 siRNA stimulated *IGFBP1* expression. Phospho-Stat3 is essential for mouse and human decidualization [38,39]. In cultured rat atrial fibroblasts, the depletion of Caveolin-1 caused the activation of STAT3 [40]. Stat5 is required for decidual differentiation of human endometrial stromal cells and contributes significantly to activation of the decidual PRL promoter [41]. Cyclooxygenase-2 (COX-2) is essential for mouse and human decidualization [42,43]. Caveolin-1 and COX-2 showed an inverse relation in colon cancer cell lines. RNAi knockdown of Caveolin-1 increased COX-2 protein level and decreased ubiquitinated COX-2 accumulation [44]. The cAMP-PKA-CREB pathway is essential to human decidualization [2]. Caveolin-1 can functionally interact with and inhibit PKA [45]. The increase of cAMP level under human decidualization also suppresses Caveolin-1 expression [46]. Based on these data, Caveolin-1 may inhibit human in vitro decidualization through suppressing the activation of Stat3, Stat5, or PKA.

### 3.4. Embryonic Regulation

In our study, Caveolin-1 signals were strongly detected in decidual cells from days 5 to 8 of pregnancy. Caveolin-1 signals were also different between delayed and activated implantation, and between day 4 of pregnancy and day 4 of pseudopregnancy. These data suggested blastocysts may be involved in Caveolin-1 regulation. Under mouse in vitro decidualization, Caveolin-1 was also upregulated. TNFα is strongly expressed and secreted by blastocysts [21]. When mouse stromal cells were treated with TNFα, Caveolin-1 expression was significantly stimulated. Different from mice, Caveolin-1 was significantly down-regulated under human in vitro decidualization. In humans, hCG is strongly produced by blastocysts and syncytiotrophoblast and mediates rescue of the corpus luteum and ensures the ongoing production of progesterone [22,23]. hCG also promotes human decidualization [23]. When human stromal cells were treated with different dosages of hCG, Caveolin-1 level was significantly suppressed by hCG. Our data from both mice and humans indicated that Caveolin-1 was regulated by blastocyst-derived molecules.

### 3.5. Insulin Regulation

Long-term type 1 diabetes impairs decidualization and extracellular matrix remodeling during early embryonic development in mice. Decreased number of implantation sites and decidual dimensions were observed in the group mated 90–110 days after diabetes induction [47]. Uterine weights were depressed in diabetic rats between days 6–7 of pseudopregnancy in association with elevated blood glucose levels (greater than 300 mg/dL) relative to control values [48].

Caveolin-1 is the central component of adipocyte caveolae and has an essential role in the regulation of insulin signaling [49]. In our study, Caveolin-1 expression was significantly stimulated by high glucose, but suppressed by insulin, suggesting that insulin-induced Caveolin-1 decrease may be beneficial for human decidualization.

## 4. Materials and Methods

### 4.1. Animals

Mature ICR mice (Sja:ICR mice, 5 weeks old) were purchased from Hunan Slack Laboratory Animal Co., LTD. All animals were housed in a specific pathogen-free (SPF) environment and maintained on a 12:12 h light-dark cycle, 22 °C temperature and 60% relative humidity.

Male ERα heterozygous KO mice on C57Bl/6J were obtained from Jackson laboratory (JAX; strain #026176, Jackson Laboratory, Bar Harbor, ME, USA) and crossed with female ICR mice to generate ERα knockout mice in ICR background. The genotypes of ERα knockout on ICR background were verified by PCR as previously described [50].

All mouse protocols were approved by the Institutional Animal Care and Use Committee of South China Agricultural University (#2019-0136). Male mice were individually caged, and female mice were caged 5 mice per cage. Pregnant mice were obtained by 1:1 mating with males of the same strain. Pseudopregnancy mice were induced by mating females with vasectomized males. Day 1 is the day of vaginal plug. The implantation sites on day 5 and day 6 were confirmed by tail vein injection of 100 μL of 1% Chicago blue dye. Mice were sacrificed at 9:00 am. Five mice were used in each group.

Ovariectomy was performed as previously described [51]. Briefly, mice were anesthetized by intraperitoneal injection with tribromoethanol at dosage of 250 mg/kg 5 min before operation. After mice were disinfected with 70% (*v/v*) ethanol, a single midline incision (0.5 cm) was cut along the back to expose the underlying muscle. The ovary was located by visualizing a white spot under the muscle. A small incision (0.5 cm) was made directly over one of the white spots. After the ovary and fat pad were removed, the oviduct and uterus were returned into the abdominal cavity. Then the muscle and skin incisions were sutured, respectively.

For hormone treatment, animals were ovariectomized and rested for two weeks. The ovariectomized mice were subcutaneously injected with progesterone (1 mg/mouse dissolved in sesame oil, Sigma-Aldrich, St. Louis, MO, USA), estradiol-17β (dissolved in sesame oil, Sigma-Aldrich), ICI 182,780 (Fulvestrant, estrogen receptor antagonist, Sigma-Aldrich), PPT (estrogen receptor α agonist, Sigma-Aldrich), and DPN (estrogen receptor β agonist, Sigma-Aldrich), respectively. Five mice were used in each group.

For delayed implantation, ten day 4 pregnant mice were ovariectomized and injected daily with 1 mg/mouse progesterone to maintain delayed implantation. Five delayed implantation mice were terminated by estradiol-17β.

### 4.2. Isolation of Mouse Uterine Endometrial Stromal Cells

Primary endometrial stromal cells were isolated and cultured as described previously [52]. Briefly, mouse uteri on day 4 of pregnancy were split and digested by 1% trypsin and 6 mg/mL dispase to remove luminal epithelial cells. The remaining uteri were incubated with 0.15 mg/mL collagenase I in 37 °C for 30 min, followed by vigorously shaking. The supernatants from the digested uteri were filtrated through 70 μm wire gauze and centrifuged to collect the stromal cells. After twice washed with the Hank’s balance salt solution, stromal cells were resuspended in complete medium (Dulbecco’s Modified Eagle’s Medium/Nutrient Mixture F-12 Ham with 2% cFBS). Stromal cells were plated onto 12-well plates at necessary concentration. In vitro decidualization was induced by 10 nM estradiol-17β plus 1 μM progesterone as previously described [53]. Stromal cells were treated with TNFα for 30 min and harvested for Western blot analysis.

### 4.3. Cell Culture and In Vitro Decidualization of Human Endometrial Stromal Cells

Immortalized human endometrial stromal cells were purchased from the American Type Culture Collection (ATCC, CRL-4003TM) and cultured according to the manuals. Briefly, human stromal cells were cultured in DMEM/F12 supplemented with 10% cFBS at 37 °C and 5% CO_2_. In vitro decidualization was performed as previously described [54]. Briefly, stromal cells were treated with 1 μM medroxyprogesterone and 0.5 mM dibutyryl cAMP to induce decidualization. Human stromal cells were treated with hCG for 12 h and harvested for Western blot analysis.

### 4.4. Caveolin-1 siRNA and Overexpression

The human Caveolin-1 siRNA sequences were synthesized as previously described [55]. The mouse Caveolin-1 siRNA was purchased from Guangzhou Riobio Co., Ltd (Guangzhou, China). A random RNA sequence not specifically for any specific genes was used as negative control (NC). The human Caveolin-1 over-expression plasmid was purchased from Hunan Fenghui Biotechnology Co., Ltd (Changsha, China). Caveolin-1 siRNA or overexpression was performed according to the instructions of the Lipofectamine 2000 kit. After transfection for 6 h, the stromal cells were induced for decidualization.

### 4.5. Real-Time PCR

Total RNAs were isolated using TRIzol reagent and reverse transcribed into cDNA with the qRT SuperMix (Vazyme). For real-time PCR, cDNA was amplified using a SYBR premix reagent on the CFX96 Touch^TM^ Real-Time system (Bio-Rad). *Rpl19* and *GAPDH* were used for normalization. *Prl8a2* (previously called *Dtprp*), *IGFBP1*, and Caveolin-1 mRNA expression were analyzed. The primer sequences used for qPCR were listed in Table 1. Data from Real-Time PCR were analyzed using the 2^−ΔΔCt^ method.

### 4.6. Western Blot Analysis

Western blot was performed as previously described [56]. Briefly, proteins from tissues or cultured cells were extracted in lysis buffer (50 mM Tris-HCl, pH 7.5, 150 mM NaCl, 1% Triton X-100, and 0.25% sodium deoxycholate). Samples were separated by 10% SDS-PAGE and transferred onto PVDF membranes. Membranes were blocked with 5% non-fat milk powder and then incubated overnight at 4 °C with each primary antibody. Primary antibodies used in this study included anti-Caveolin-1 (sc-894, Santa Cruz, CA, USA), anti-GAPHD (sc-25778, Santa Cruz), anti-a-Tubulin (2144, Cell Signaling Technology), anti-p-CREB (9198s, Cell Signaling Technology, Danvers, MA, USA), anti-p53 (2521p, Cell Signaling Technology), anti-p21 (10355-1-AP, Proteintech, Wuhan, China), anti-ZO1 (ab221547, Abcam, Cambridge, UK). The membranes were incubated with HRP-conjugated secondary antibody at room temperature for 1 h. ECL chemiluminescent kits (Milipore) were used for detecting the signals.

### 4.7. Immunofluorescence

Frozen sections (10 μm) were processed for immunofluorescence as described previously [57]. Briefly, the sections were fixed in 4% FPA in PBS, permeabilized by 0.1% Triton X-100 in PBS for 15 min, and blocked with 10% normal goat serum for 40 min. The sections were incubated with anti-Caveolin-1 primary antibody overnight at 4 °C. Subsequently, the sections were incubated with appropriate fluorescent dye-conjugated secondary antibody and counterstained with DPAI.

### 4.8. SA-β-Gal Staining

Staining of SA-β-gal activity was performed as described previously [58]. Cells were fixed in 0.5% glutaraldehyde in PBS and stained for overnight in PBS (pH 6.0) containing 1 mM MgCl_2_, 1 mg/mL X-gal, and 5 mM each of potassium ferricyanide and potassium ferrocyanide.

### 4.9. Statistical Analysis

Experiments in this study were repeated at least three times independently. Five mice were used in each group. The differences between the two groups were compared by Student’s t-test. Multiple comparisons were performed using the one-way ANOVA test followed by the Newman–Keuls test. *p* < 0.05 was considered to be statistically significant. Post hoc power analysis was performed by using G*Power. The powers of all the data in this study were more than 0.95.

## 5. Conclusions

Our data indicated that Caveolin-1 is strongly expressed in mouse uterus during early pregnancy and differentially regulated by estrogen and progesterone. Blastocysts-derived TNFα and hCG regulate Caveolin-1 in mouse and human decidual cells, respectively. A low level of Caveolin-1 should be beneficial for human decidualization. Data from this study should shed lights on understanding the underlying mechanism during decidualization and diagnosing decidualization failure.

## Figures and Tables

**Table 1:** Primers and siRNA sequences used in this study.

Gene	Species	Sequence (5′-3′)	Application	Accession Number	Product Size (bp)
*Rpl7*	Mouse	GCAGATGTACCGCACTGAGATTC ACCTTTGGGCTTACTCCATTGATA	RT-qPCR	NM_011291.5	129
*Prl8a2*	Mouse	AGCCAGAAATCACTGCCACT TGATCCATGCACCCATAAAA	RT-qPCR	NM_010088	119
*Rpl19*	Mouse	TCATGGAGCACATCCACAAGCTGA CGCTTTCGTGCTTCCTTGGTCTTA	RT-qPCR	NM_001287738.1	197
*Cav1*	Mouse	ATTCAGCAACATCCGCATCAG AGAGTGAGGACAGCAACCAAT	RT-qPCR	NM_001243064.1	494
*IGFBP1*	Human	CCAAACTGCAACAAGAATG GTAGACGCACCAGCAGAG	RT-qPCR	NM_001013029	87
*RPL7*	Human	CTGCTGTGCCAGAAACCCTT TCTTGCCATCCTCGCCAT	RT-qPCR	NM_000971	194
*GAPDH*	Human	GAAGGTGAAGGTCGGAGT GATGGCAACAATATCCACTT	RT-qPCR	BC023632	94
*CAV1*	Human	GCATCAGCCGTGTCTATTCC GCAGTTGAGGTTGTTGGTTCT	RT-qPCR	NM_001172896.2	476
*Cav1*	Mouse	CCACCTTCACTGTGACAAA	siRNA		
*CAV1*	Human	GCCGUGUCUAUUCCAUCUA	siRNA		
*NC*	-	CTCCGAACGTGTCACGT	siRNA		

## Data Availability

All the data generated in this study are included in this manuscript.

## Abbreviations

CAV1	Caveolin-1
COX2	Cyclooxygenase-2
CREB	cAMP-response element-binding protein
DPN	Diarylpropionitrile
ERα	Estrogen receptor α
hCG	human chorionic gonadotropin
ICI	ICI 182780 (Fulvestrant)
PPT	Estrogen receptor α agonist
SA-β-Gal	Senescence-associated-β-galactosidase
PR	Progesterone receptor
PRL	Prolactin
TNFα	Tumor necrosis factor α
ZO-1	Zonula occludens-1
